# Prevalence of Adjustment Disorder and Its Predictors Among First- and Second-Year Medical Students in Madinah, Saudi Arabia

**DOI:** 10.7759/cureus.52028

**Published:** 2024-01-10

**Authors:** Nadir Makki, Dalia A Alrehaili, Renad K Alrehaili, Rawan Sedaqir, Weam T Alahmadi

**Affiliations:** 1 Psychiatry, Taibah University, Madinah, SAU; 2 Medical School, Taibah University, Madinah, SAU

**Keywords:** stress, too much study, adnm-20, medical students, adjustment disorder

## Abstract

Background

The first years of college, notably the first and second, are challenging and time-intensive, frequently characterized by substantial pressure that can lead to dissatisfaction among new students. Such an environment may precipitate adjustment difficulties, potentially resulting in depression, anxiety, and stress. This phenomenon is particularly pronounced among medical students. Despite the widespread nature of these challenges, research focusing on the prevalence of adjustment disorder among medical students in Madinah, Saudi Arabia, is notably scarce in the literature. This study aims to evaluate the prevalence and identify predictors of adjustment disorder among a substantial cohort of first- and second-year medical students in Madinah.

Methodology

An institution-based, quantitative, cross-sectional study was conducted from April 2022 to August 2022. The study encompassed a total of 273 first- and second-year medical students from the Madinah region. To measure stressor exposure and symptoms of adjustment disorder, the Adjustment Disorder-New Module 20 (ADNM-20) scale was employed.

Results

Our study, comprising 273 participants from Taibah University and Al-Rayan Colleges, revealed a higher prevalence of adjustment disorder among Al-Rayan students: 54.8% (n = 63) compared to their counterparts at Taibah University 41.8% (n = 66), with a p-value of 0.033. However, no significant association was found with other demographic factors. Utilizing the ADNM-20 questionnaire, the study identified prevalent symptoms of adjustment disorder among participants. Key findings included 47.6% (n = 130) of participants feeling low and sad, 41% (n = 112) experiencing repetitive stressful thoughts, approximately one-third (n = 81) exhibiting avoidance behaviors and intrusive thoughts, 38.1% (n = 104) reducing enjoyable activities, 29.3% (n = 80) encountering increased anxiety, 30% (n = 80) reporting irritability, 31.1% (n = 85) facing concentration issues, 23.4% (n = 64) having sleep disturbances, and 28.2% (n = 77) observing impacts on personal and leisure activities.

Conclusions

The study concludes that adjustment disorder is prevalent among new university students, particularly at Al-Rayan University, where it manifests in various symptoms including mood disturbances and anxiety.

## Introduction

Transitioning from high school to college constitutes a significant, stressful, social, and psychological event for first-year students [1]. While entering college is generally regarded as a positive milestone, it can challenge an individual’s sense of personal security, physical comfort, and capacity for enjoying gratifying activities [2]. The initial years at the university, particularly for medical students compared to other specialties, are notably challenging. These years demand a heightened effort from students to adapt to psychological pressures. Adjustment is a psychological process that aids in managing stress and is crucial for enhancing students’ academic performance [3].

In the college environment, students encounter developmental challenges in unfamiliar and often demanding situations. The initial stages of this transition, especially the struggles faced by first-year students, are emphasized in research, as noted by Bruffaerts et al. (2018) [4]. How students respond to these stressors can have significant and long-term consequences [5].

Adjustment disorder, as defined by the Diagnostic and Statistical Manual of Mental Health 5, is the development of emotional or behavioral symptoms in response to an identifiable stressor occurring within three months of the onset of the stressor [6]. Among university students, it can stem from numerous factors impacting mental health, particularly within the first three months of exposure to stress. Key factors, as identified by prior research, include separation from family and friends, changes in the living environment, difficulties in forming new friendships, emotional and financial crises, health concerns, and challenges in time management [7]. Studies among young adults indicate that those living with their parents are less prone to emotional issues and tend to have behaviors more influenced by parental guidance [8]. Recently, the COVID-19 pandemic has emerged as a factor exacerbating adjustment disorders among medical students [9]. These factors can deteriorate students’ academic performance and mental health, increasing their susceptibility to anxiety, stress, and depression [3]. Despite the prevalence of these issues, there is a notable dearth of research on this topic in Saudi Arabia. Consequently, this study aims to assess the prevalence of adjustment disorder and its associated factors among university students.

## Materials and methods

This study was designed as an observational cross-sectional study conducted among first- and second-year medical students at Taibah University and Al-Rayan University in Madinah, Saudi Arabia. It excluded medical students from other years and first- and second-year medical students not located in Madinah. Data collection occurred from April to August 2022.

Ethical approval was granted by the Scientific Ethical Committee at Taibah University. Participants were informed of the voluntary nature of their involvement, with assurances of confidentiality, and that responses would be used solely for scientific purposes. They were given the option to consent or to decline participation.

The combined student population at both universities for the two years totaled 573. Using the OpenEpi sample size calculator [10], we determined that a sample size of 231 was necessary, accounting for a 5% margin of error and a 95% confidence level. To accommodate potential non-responses, this number was increased by 20%, resulting in a total of 277.

The questionnaire was distributed via the colleges’ WhatsApp groups, inviting students to participate in a voluntary web-based survey. Consent was obtained before participation, and we received 273 responses out of 573 students through Google Forms, with a response rate of 48%.

The questionnaire comprised two main sections. The first section included sociodemographic factors (age, gender, academic year, marital status, living situation, and monthly income). The second section utilized the Adjustment Disorder-New Module 20 (ADNM-20) scale, a psychological assessment tool validated by Glaesmer et al. [11]. This self-administered instrument consists of two parts, namely, a list of acute and chronic life events from the past two years, and items assessing symptoms related to the most significant of these events. The scale facilitates a comprehensive assessment of stressors and their psychological effects. Comprising 20 items, it was used in its original English format, as participants are proficient in English due to their English-based educational curriculum. For this study, 10 relevant stressors were selected, i.e., divorce or separation, family conflict, illness in the family, death of a loved one, academic overload, deadline pressures, relocating, financial difficulties, personal illness, and ceasing a key leisure activity. The symptom section of the questionnaire includes 20 questions, categorized into core symptoms (preoccupation with four items, failure to adapt with four items) and accessory symptoms (avoidance with four items, depressive mood with three items, anxiety with two items, and impulse disturbance with three items). Participants rated the frequency of their reactions to the stressors on a scale from 1 (never) to 4 (often) and noted the duration of these reactions, ranging from less than a month to up to two years. Of note, in the questionnaire and results table, the years were referred to as second and third because of a preparatory year before enrolling in medical college. Therefore, the second and third years of university education correspond to the first and second years of medical college.

We performed a pilot study involving 10 students, randomly selected from two universities. The primary aim of this preliminary study was twofold: first, to verify the clarity, specificity, and neutrality of the demographic questions we developed; and second, to ascertain the time participants would typically require to complete the questionnaire. This questionnaire was disseminated to the participants via WhatsApp. Feedback was meticulously gathered from each of the 10 participants individually. It is important to note that the data obtained from this pilot study were not included in the final analysis of our main study, as their primary purpose was to fine-tune our research tools and procedures. During this pilot phase, none of the participants raised significant concerns or offered substantial comments about the questionnaire, suggesting its initial effectiveness and suitability. To enhance the credibility of this pilot phase, a consultant psychiatrist oversaw the process, lending their expertise to ensure both the face validity and accuracy of the pilot study. We did not deem it necessary to conduct further validity tests. This decision was underpinned by our use of a scale that had already been validated, thus providing a strong foundation of reliability for our research instrument.

After data collection, it was checked for accuracy, completeness, and consistency. The data were then coded and entered into SPSS version 23 (IBM Corp., Armonk, NY, USA). Frequency and percentages were used to present categorical variables. The chi-square test was employed to evaluate associations between categorical variables, with the level of significance set at 0.05.

## Results

**Figure 1:**
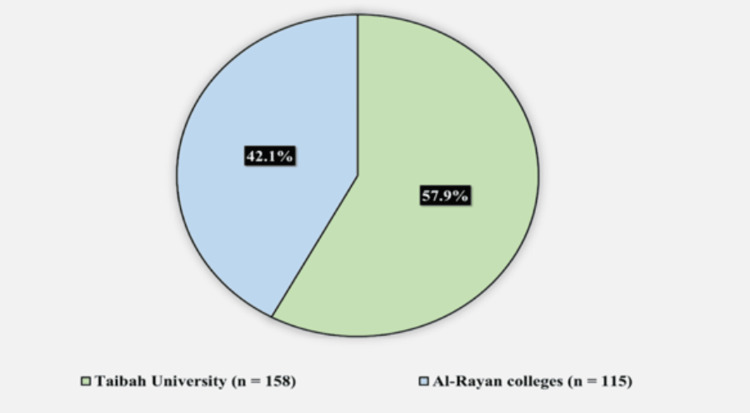
Participants’ college (n = 273). The data are represented as N with its corresponding %.

In the study, a total of 273 participants were included, of whom 158 (57.9%) were from Taibah University, and 115 (42.1%) were from Al-Rayan Colleges (Figure 1).

The demographic characteristics of the participants revealed that a significant proportion, 34.1% or 93 individuals, were aged 18 to 20, while the majority, 64.1% or 175 respondents, fell within the 21 to 23 age brackets, and a minor segment, 1.8% or five participants, were 24 years or older. Regarding gender distribution, females represented 60.1% with 164 respondents, while males comprised 39.9% with 109 respondents. Regarding academic standing, 59.3% or 162 respondents were third-year students, whereas 40.7% or 111 were in their second year. A predominant majority, 99.3% or 271 participants, were unmarried, contrasting with a mere 0.7% or two respondents who were married. Residential patterns indicated that most, 89.4% or 244 respondents, resided with family, while 8.4% or 23 lived independently, and a small fraction, 2.2% or six, stayed on campus. In terms of monthly income, a vast majority, 90.8% or 248 students, reported earning below 5,000 Saudi Riyals, whereas a smaller proportion, 9.2% or 25 students, earned between 5,000 and 10,000 Saudi Riyals, as detailed in Table 1.

**Table 1:** Sociodemographic profile of the participants (n = 273). The data are represented as N with its corresponding %.

Demographic characteristics	n	%
Age		
18–20 years	93	34.10
21–23 years	175	64.10
24 years and older	5	1.80
Gender		
Male	109	39.90
Female	164	60.10
Academic year		
Second year	111	40.70
Third year	162	59.30
Marital status		
Single	271	99.30
Married	2	0.70
Living situation		
Lives independently	23	8.40
Lives in campus	6	2.20
Lives with family	244	89.40
Monthly income		
Less than 5,000 SR	248	90.80
5,000–10,000 SR	25	9.20

Adjustment disorder results among our sample

**Figure 2:**
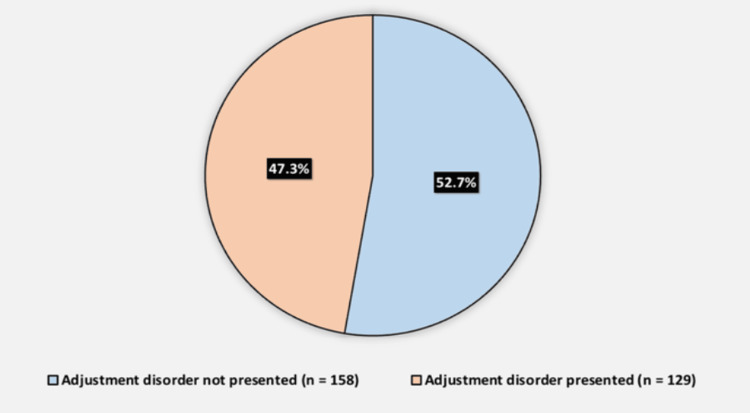
Prevalence of adjustment disorder among participants. The data are represented as n with its corresponding %.

Of the participants, 129 (47.3%) were identified with adjustment disorder, while 158 (52.7%) did not exhibit the disorder (Figure 2).

**Figure 3:**
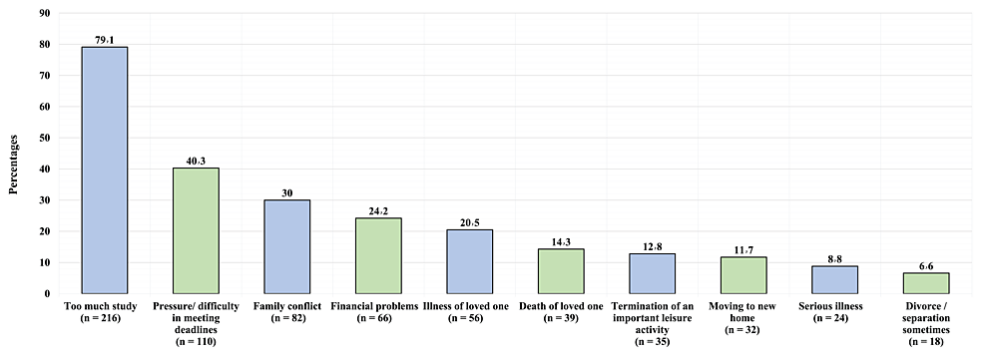
Participants’ experience with stressful events. The data are presented as a percentage above the columns with its number below the column.

In our study, the personal narratives of the participants painted a vivid picture of the challenges they faced. A substantial 79.1% (216 participants) shared their struggles with the overwhelming demands of their academic workload. For 40.3% (110 participants), the race against time to meet pressing deadlines was a significant source of stress. The complexities of family dynamics were not far behind, affecting 30% (82 participants) who navigated through family conflicts. Financial constraints posed a concern for 24.2% (66 participants), underscoring the often-overlooked economic pressures faced by students. The emotional toll of illness of loved ones was a reality for 20.5% (56 participants), highlighting the intersection of personal life and academic responsibilities. The grief of losing loved ones was an unfortunate reality for 14.3% (39 participants), a poignant reminder of the personal losses that often go unseen in academic settings. The need to sacrifice cherished leisure activities was felt by 12.8% (35 participants), illustrating the trade-offs students make in pursuit of their studies. Life transitions, such as moving to a new home, presented challenges for 11.7% (32 participants), while dealing with personal health issues was a concern for 8.8% (24 participants). Furthermore, navigating the emotional landscape of divorce or separation was a challenge faced by 6.6% (18 participants), highlighting the diverse and complex issues that university students encounter (Figure 3).

Factors related to adjustment disorder

In our exploration of factors related to adjustment disorder, we discovered a notable trend tied to the educational institutions of the participants. At Al-Rayan University, students faced a considerably higher incidence of adjustment disorder, with 54.8% (n = 63) reporting such challenges, in contrast to 41.8% (n = 66) at Taibah University (p = 0.033). This finding points to the unique environments of different colleges influencing student well-being. However, when considering other factors such as age, gender, academic year, marital status, living arrangements, and monthly income, there was no significant link with the likelihood of experiencing adjustment disorder, as detailed in Table 2.

**Table 2:** Factors associated with the presence of adjustment disorder. The data are represented as n with its corresponding %. P-value is considered significant at <0.05.

Factor	Prevalence of adjustment disorder		Chi-square value	Degrees of Freedom	P-value
	No	Yes			
Age			2.317	2	0.314
18–20 years	51 (54.8%)	42 (45.2%)			
21–23 years	92 (52.6%)	83 (47.4%)			
24 years and older	1 (20%)	4 (80%)			
Gender			3.452	1	0.063
Male	65 (59.6%)	44 (40.4%)			
Female	79 (48.2%)	85 (51.8%)			
Academic year			1.810	1	0.179
Second year	64 (57.7%)	47 (42.3%)			
Third year	80 (49.4%)	82 (50.6%)			
Marital status			1.805	1	0.179
Single	142 (52.4%)	129 (47.6%)			
Married	2 (100%)	2 (0%)			
Living situation			2.854	1	0.240
Lives independent	16 (69.6%)	7 (30.4%)			
Lives in campus	3 (50%)	3 (50%)			
Lives with family	125 (51.2%)	119 (48.8%)			
Monthly income			2.569	1	0.109
Less than 5,000 SR	127 (51.2%)	121 (48.8%)			
5,000–10,000 SR	17 (68%)	8 (32%)			
College			4.520	1	0.033*
Taibah University	92 (58.2%)	66 (41.8%)			
Al-Rayan Colleges	52 (45.2%)	63 (54.8%)			

Concerning participant responses to the ADNM-20 questionnaire, Table 3 presents a comprehensive list of all identified stressful events, along with the frequency of their occurrence in the sample.

**Table 3:** Participants’ responses toward Adjustment Disorder-New Module 20 questionnaire (n = 273). The data are represented as n with its corresponding %.

Question	n	%	Question	n	%
Q1: Since the stressful problem, I feel low and sad			Duration of complaint		
Never	29	10.6	Never	29	10.6
Rarely	42	15.4	<1 month	128	46.9
Sometimes	130	47.6	1 to 6 months	69	25.3
Often	72	26.4	6 months to 2 years	47	17.2
Q2: I have to think about the stressful situation repeatedly			Duration of complaint		
Never	33	12.1	Never	33	12.1
Rarely	56	20.5	<1 month	117	42.9
Sometimes	112	41	1 to 6 months	71	26
Often	72	26.4	6 months to 2 years	52	19
Q3: I try to avoid talking about the stressful situation wherever possible			Duration of complaint		
Never	51	18.7	Never	51	18.7
Rarely	44	16.1	<1 month	109	39.9
Sometimes	85	31.1	1 to 6 months	57	20.9
Often	93	34.1	6 months to 2 years	56	20.5
Q4: I keep having to think about the stressful situation and this is a great burden to me			Duration of complaint		
Never	53	19.4	Never	53	19.4
Rarely	60	22	<1 month	101	37
Sometimes	102	37.4	1 to 6 months	67	24.5
Often	58	21.2	6 months to 2 years	52	19
Q5: Nowadays, I do those activities which I used to enjoy much more rarely			Duration of complaint		
Never	60	22	Never	60	22
Rarely	51	18.7	<1 month	92	33.7
Sometimes	104	38.1	1 to 6 months	76	27.8
Often	58	21.2	6 months to 2 years	45	16.5
Q6: If I think about the stressful situation, I find myself in a real state of anxiety			Duration of complaint		
Never	47	17.2	Never	47	17.2
Rarely	66	24.2	<1 month	97	35.5
Sometimes	80	29.3	1 to 6 months	71	26
Often	80	29.3	6 months to 2 years	58	21.2
Q7: I avoid certain things that might remind me of the stressful situation			Duration of complaint		
Never	51	18.7	Never	51	18.7
Rarely	52	19	<1 month	102	37.4
Sometimes	101	37	1 to 6 months	68	24.9
Often	69	25.3	6 months to 2 years	52	19
Q8: I am nervous and restless since the stressful situation			Duration of complaint		
Never	57	20.9	Never	57	20.9
Rarely	70	25.6	<1 month	94	34.4
Sometimes	85	31.1	1 to 6 months	63	23.1
Often	61	22.3	6 months to 2 years	59	21.6
Q9: Since the stressful situation, I am much quicker to lose my temper, even over small things			Duration of complaint		
Never	71	26	Never	71	26
Rarely	73	26.7	<1 month	85	31.1
Sometimes	75	27.5	1 to 6 months	59	21.6
Often	54	19.8	6 months to 2 years	58	21.2
Q10: Since the stressful situation, I can only concentrate on certain things with difficulty			Duration of complaint		
Never	74	27.1	Never	74	27.1
Rarely	61	22.3	<1 month	77	28.2
Sometimes	85	31.1	1 to 6 months	67	24.5
Often	53	19.4	6 months to 2 years	55	20.1
Q11: I try to abolish the stressful situation from my memory			Duration of complaint		
Never	40	14.7	Never	40	14.7
Rarely	61	22.3	<1 month	103	37.7
Sometimes	98	35.9	1 to 6 months	76	27.8
Often	74	27.1	6 months to 2 years	54	19.8
Q12: I have noticed that I am becoming more irritable due to the stressful situation			Duration of complaint		
Never	63	23.1	Never	63	23.1
Rarely	67	24.5	<1 month	97	35.5
Sometimes	82	30	1 to 6 months	62	22.7
Often	61	22.3	6 months to 2 years	51	18.7
Q13: I get constant memories of the stressful situation and can’t do thing to stop them			Duration of complaint		
Never	71	26	Never	71	26
Rarely	66	24.2	< 1 month	73	26.7
Sometimes	75	27.5	1 to 6 months	67	24.5
Often	61	22.3	6 months to 2 years	62	22.7
Q14: I try to suppress my feelings because they are a burden to me			Duration of complaint		
Never	65	23.8	Never	65	23.8
Rarely	65	23.8	<1 month	79	28.9
Sometimes	85	31.1	1 to 6 months	73	26.7
Often	58	21.2	6 months to 2 years	56	20.5
Q15: My thoughts revolve around anything to do with the stressful situation			Duration of complaint		
Never	77	28.2	Never	77	28.2
Rarely	72	26.4	<1 month	85	31.1
Sometimes	83	30.4	1 to 6 months	68	24.9
Often	41	15	6 months to 2 years	43	15.8
Q16: Since the stressful situation, I am scared of doing certain things or of getting into certain situations			Duration of complaint		
Never	86	31.5	Never	86	31.5
Rarely	63	23.1	<1 month	70	25.6
Sometimes	70	25.6	1 to 6 months	70	25.6
Often	54	19.8	6 months to 2 years	47	17.2
Q17: Since the stressful situation, I don’t like going to work or carrying out the necessary tasks in everyday life			Duration of complaint		
Never	99	36.3	Never	99	36.3
Rarely	64	23.4	<1 month	74	27.1
Sometimes	69	25.3	1 to 6 months	58	21.2
Often	41	15	6 months to 2 years	42	15.4
Q18: I have been feeling dispirited since the stressful situation and have little hope for the future			Duration of complaint		
Never	96	35.2	Never	96	35.2
Rarely	66	24.2	<1 month	71	26
Sometimes	70	25.6	1 to 6 months	61	22.3
Often	41	15	6 months to 2 years	45	16.5
Q19: Since the stressful situation, I can no longer sleep properly			Duration of complaint		
Never	91	33.3	Never	91	33.3
Rarely	75	27.5	<1 month	85	31.1
Sometimes	64	23.4	1 to 6 months	55	20.1
Often	43	15.8	6 months to 2 years	42	15.4
Q20: Overall, the situation affected me strongly in my personal relationships, my leisure activities, or other important areas of my life			Duration of complaint		
Never	62	22.7	Never	62	22.7
Rarely	82	30	<1 month	83	30.4
Sometimes	77	28.2	1 to 6 months	73	26.7
Often	52	19	6 months to 2 years	55	20.1

Regarding the frequency of symptoms, a considerable proportion of participants frequently reported symptoms associated with adjustment disorder. Notably, 47.6% of respondents occasionally experienced feelings of sadness and low mood (Q1), and 41% intermittently dwelled on the stressful situation (Q2). This reflects a moderate level of distress among a significant segment of the participants.

Regarding avoidance and intrusive thoughts, the data revealed a prominent pattern in avoidance behaviors and intrusive thoughts. In response to Q3, 31.1% of participants occasionally tried to avoid discussing the stressful situation, while 34.1% often did so. Similarly, Q4 responses indicated that 37.4% occasionally were plagued by persistent thoughts about the stressor. These findings highlight a widespread struggle with avoidance and intrusive thoughts.

Regarding the impact on enjoyment and anxiety, responses to Q5 and Q6 demonstrated a substantial effect on leisure activities and an increase in anxiety. Specifically, 38.1% of participants engaged less in previously enjoyable activities, and 29.3% frequently felt anxious about the stressful situation.

The duration of symptoms varied among respondents. For most items, the majority reported experiencing symptoms for less than a month. However, a considerable number experienced symptoms for longer periods, underscoring the potential chronic nature of adjustment disorders.

Regarding irritability and concentration issues, there were notable reports of increased irritability (Q12) and difficulties with concentration (Q10), with 30% and 31.1% of participants occasionally encountering these issues, respectively. This suggests that adjustment disorder can markedly impact emotional regulation and cognitive function.

Concerns regarding sleep disturbances (Q19) and the impact on personal relationships and leisure activities (Q20) were also significant. A total of 33.3% experienced sleep-related issues and 28.2% observed a pronounced effect on their personal and leisure lives.

Comparison of participants’ experience with stressful events across colleges

**Figure 4:**
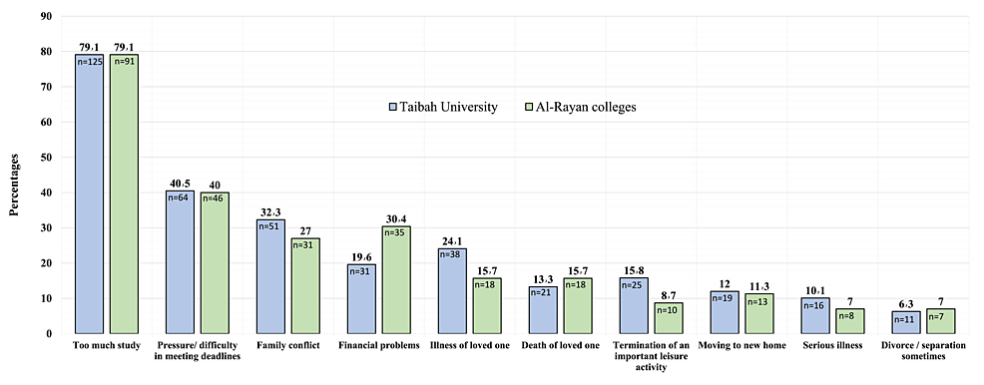
Comparison of participants’ experience with stressful events across college. The data is represented as a percentage above the column with its corresponding number within the same column.

A similar pattern of stressful events was observed between the two colleges, corresponding to the general pattern of stressful life events with no significant differences between participants from either institution. The sole exception to this was financial problems, where a notable disparity emerged. Participants from Al-Rayan Colleges reported a significantly higher incidence of financial difficulties compared to those from Taibah University (30.4% vs. 19.6%, p = 0.039), as illustrated in Figure 4.

## Discussion

As previously established, the path to medical education and health-related professions is incredibly stressful, particularly for recent high school graduates (first- and second-year medical students) [12]. The training involves various stressors, such as adapting to a high workload within limited timeframes, learning a new language for non-native speakers, familiarizing with medical terminology, memorizing vast amounts of information, frequent evaluations, peer competitiveness, financial constraints, and limited time for recreation and social relationships [13]. Consequently, medical students, especially in their first and second years, face a heightened risk of psychological distress and illness, struggling more to adjust to these stressors compared to non-medical peers [13-16]. Prolonged stress can lead to various mental illnesses, including depression, anxiety, and adjustment disorder [17,18]. According to the International Classification of Diseases 11th Revision, adjustment disorder is a maladaptive reaction to identifiable psychosocial stressors, typically occurring within a month of the stressor [19]. While outside the scope of our study, research indicates that patients with adjustment disorder are more prone to substance abuse and impulsive behaviors [20], with an increased risk of suicidal acts, particularly among adolescents [21-23].

The prevalence of adjustment disorder among first- and second-year medical students in this survey was 47.3%, highlighting significant psychological distress and low resilience against stressors. This rate is consistent with other studies among university students. For example, studies in Ethiopia reported adjustment disorder rates of 42.5% and 48% among first-year undergraduate students at Dilla and Jimma Universities, respectively [7,24]. Additionally, a study at Gulf University in Bahrain found a 36.8% prevalence of adjustment disorder among medical students, particularly first-year students [25]. Similar trends were seen in North Jordan and Malaysia, with rates of 42.8% and 50%, respectively [26,27]. However, a study in Riyadh reported a lower rate of 21.3%, possibly due to early-year assessments before exposure to significant stressors [28]. A more comprehensive, nationwide survey across all universities and colleges in Saudi Arabia is necessary for a more accurate assessment of adjustment disorder.

In this study, the most reported stressors were excessive studying (79.1%), pressure and difficulty meeting deadlines (40.3%), financial issues, illness in loved ones, and bereavement (24.2%, 20.5%, and 14.3%, respectively). These findings align with a study from Riyadh, Saudi Arabia, among first-year medical students, but contrast with a study at Duhok University in Iraq, where personal illness or that of a loved one and family conflicts were the main stressors [28,29]. The similarities with Riyadh and differences with Iraq may be attributed to shared cultural, social, and environmental values.

In this survey, the only significant factor associated with adjustment disorder was the participant’s college. Students from Al-Rayan University had a higher rate of adjustment disorder compared to Taibah University, potentially linked to greater financial challenges at Al-Rayan. Conversely, a study in Riyadh found gender to be a significant predictor, with female students at higher risk [28]. Age did not show a significant association with AD in this study, aligning with findings from King Saud University that indicated an inverse relationship between age and adjustment disorder risk, emphasizing the vulnerability of younger medical students [28].

Strengths and limitations

This contribution of this study is significant, focusing on a crucial issue impacting the mental health of aspiring doctors. It appears to fill a notable gap in the current body of research, as there seems to be a scarcity of studies addressing the prevalence of adjustment disorder among early-year medical students, both in Saudi Arabia and globally. By examining this specific demographic in the Madinah region, our research adds valuable insights to the literature on their psychological health and adjustment disorder prevalence. Nonetheless, it is pertinent to note certain limitations, including the methodology, involving a cross-sectional design, and self-report questionnaires, which might somewhat limit the precision of our findings.

## Conclusions

Adjustment disorder was prevalent in our sample, and even more so among students at Al-Rayan University. Our sample exhibited various symptoms, such as mood disturbances and anxiety. These findings advocate for further research to understand the causes of this prevalence in student groups, as well as to enhance prevention and treatment methods.

## Appendices

Questionnaire to assess the prevalence of adjustment disorder and its predictors among first- and second-year medical students in Madinah, Saudi Arabia

First part

**Table 4:** Socioeconomic data.

Socioeconomic data	
Gender	Female
	Male
Age	18–20
	21–23
	24 years and older
College	Taibah University
	Al-Rayan Colleges
Academic year	2nd year (first medical year)
	3rd year (second medical year)
Marital situation	Single
	Married
	Divorced
	Widow
Living situation	Lives independent
	Lives in campus
	Lives with family
Are you originally from the Madinah region?	Yes
	No

Second part

Below is a list of stressful life events. Please indicate those events that happened during the past year and are currently a strong burden to you, or have burdened you in the last six months. You can indicate as many events as applicable.

**Table 5:** Section 2: list of stressful events.

Yes	
	01. Divorce/separation Sometimes
	02. Family conflict
	03. Illness of a loved one
	04. Death of a loved one
	05. Too much study
	06. Pressure/Difficulty in meeting deadlines
	07. Moving to a new home
	08. Financial problems
	09. Own serious illness
	10. Termination of an important leisure activity

Third part

In the following, you will find various statements about which reactions these types of events can trigger. In the first step, we ask you to indicate how often the respective statement applies to you (“never” to “often”).

In the second step, we would like to ask you to indicate how long you have been having this reaction. It can be less than one month (<1 month), for approximately one month to half a year (<6 months), or longer than 6 months (>6 months).

This will probably not be very easy to estimate, but please try to give a rough classification of the duration of the reaction.

**Table 6:** Adjustment Disorders-New Module 20.

Question	Frequency in the last week	Duration of complaint
1: Since the stressful problem, I feel low and sad	Never, Rarely, Sometimes, Often	Less than 1 month, 1 to 6 months, 6 months to 2 years
2: I have to think about the stressful situation repeatedly	Never, Rarely, Sometimes, Often	Less than 1 month, 1 to 6 months, 6 months to 2 years
3: I try to avoid talking about the stressful situation wherever possible	Never, Rarely, Sometimes, Often	Less than 1 month, 1 to 6 months, 6 months to 2 years
4: I keep having to think about the stressful situation and this is a great burden to me	Never, Rarely, Sometimes, Often	Less than 1 month, 1 to 6 months, 6 months to 2 years
5: Nowadays, I do those activities which I used to enjoy much more rarely	Never, Rarely, Sometimes, Often	Less than 1 month, 1 to 6 months, 6 months to 2 years
6: If I think about the stressful situation, I find myself in a real state of anxiety	Never, Rarely, Sometimes, Often	Less than 1 month, 1 to 6 months, 6 months to 2 years
7: I avoid certain things that might remind me of the stressful situation	Never, Rarely, Sometimes, Often	Less than 1 month, 1 to 6 months, 6 months to 2 years
8: I am nervous and restless since the stressful situation	Never, Rarely, Sometimes, Often	Less than 1 month, 1 to 6 months, 6 months to 2 years
9: Since the stressful situation, I am much quicker to lose my temper, even over small things	Never, Rarely, Sometimes, Often	Less than 1 month, 1 to 6 months, 6 months to 2 years
10: Since the stressful situation, I can only concentrate on certain things with difficulty	Never, Rarely, Sometimes, Often	Less than 1 month, 1 to 6 months, 6 months to 2 years
11: I try to abolish the stressful situation from my memory	Never, Rarely, Sometimes, Often	Less than 1 month, 1 to 6 months, 6 months to 2 years
12: I have noticed that I am becoming more irritable due to the stressful situation	Never, Rarely, Sometimes, Often	Less than 1 month, 1 to 6 months, 6 months to 2 years
13: I get constant memories of the stressful situation and can’t do anything to stop them	Never, Rarely, Sometimes, Often	Less than 1 month, 1 to 6 months, 6 months to 2 years
14: I try to suppress my feelings because they are a burden to me	Never, Rarely, Sometimes, Often	Less than 1 month, 1 to 6 months, 6 months to 2 years
15: My thoughts revolve around anything to do with the stressful situation	Never, Rarely, Sometimes, Often	Less than 1 month, 1 to 6 months, 6 months to 2 years
16: Since the stressful situation, I am scared of doing certain thing or of getting into certain situations	Never, Rarely, Sometimes, Often	Less than 1 month, 1 to 6 months, 6 months to 2 years
17: Since the stressful situation, I don’t like going to work or carrying out the necessary tasks in everyday life	Never, Rarely, Sometimes, Often	Less than 1 month, 1 to 6 months, 6 months to 2 years
18: I have been feeling dispirited since the stressful situation and have little hope for the future	Never, Rarely, Sometimes, Often	Less than 1 month, 1 to 6 months, 6 months to 2 years
19: Since the stressful situation, I can no longer sleep properly	Never, Rarely, Sometimes, Often	Less than 1 month, 1 to 6 months, 6 months to 2 years
20: Overall, the situation affected me strongly in my personal relationships, my leisure activities, or other important areas of my life	Never, Rarely, Sometimes, Often	Less than 1 month, 1 to 6 months, 6 months to 2 years
